# Diagnostic Utility of Fine-Needle Aspiration Cytology (FNAC) and Frozen Section Against Histopathology in Evaluating Benign and Malignant Breast Lesions

**DOI:** 10.7759/cureus.53108

**Published:** 2024-01-28

**Authors:** Amrita D Sharma, Kartikeya Ojha, Navya B N

**Affiliations:** 1 Pathology, Central Referral Hospital, Sikkim Manipal Institute of Medical Sciences, Gangtok, IND; 2 Internal Medicine, Central Referral Hospital, Sikkim Manipal Institute of Medical Sciences, Gangtok, IND; 3 Pathology, KVG Medical College & Hospital, Sullia, IND

**Keywords:** histopathology, frozen section, fnac, breast lesions, breast carcinoma

## Abstract

Introduction: Breast lesions, particularly lumps, pose concerns for females, varying between benign and malignant conditions. Accurate differentiation solely through clinical assessment is challenging, necessitating a definitive diagnostic strategy. Fine-needle aspiration cytology (FNAC) is integral in the "triple approach" to breast evaluation, offering simplicity, reliability, and cost-effectiveness. However, FNAC has limitations, occasionally failing to yield definitive diagnoses due to inherent constraints. Contrarily, frozen-section analysis, a long-standing intraoperative diagnostic method, plays a crucial role in swift diagnosis during surgeries. Despite technological advancements, frozen sections serve specific diagnostic purposes, confirming carcinoma when FNAC is inconclusive and evaluating resected margins. However, freezing artifacts may affect tissue assessment, emphasizing the continued reliance on histopathology for guiding treatment decisions.

Objectives: This study was conducted at KVG Medical College and Hospital, Sullia, Karnataka, India. It aimed to analyze the morphological characteristics of benign and malignant breast lesions using FNAC, frozen section, and histopathology and evaluate the sensitivity, specificity, and predictive values of FNAC and frozen section against histopathology as the reference standard.

Methods: A cross-sectional investigation was carried out at a tertiary care hospital's Department of Pathology, on 60 female patients who presented with palpable breast masses over a span of two and a half years. FNAC was conducted, and the observations were classified into five categories as per the International Academy of Cytology guidelines. In addition, intraoperative frozen-section analysis was undertaken. A comparative analysis was conducted between the FNAC and intraoperative frozen-section findings, juxtaposed with the subsequent histopathological diagnoses.

Results: FNAC revealed 51.7% malignant, 45% benign, and 3.3% inadequate cases; the frozen-section analysis indicated 51.6% malignant, 45% benign, and 3.3% deferred cases; histopathology showed 53.3% malignant, 45% benign, and 1.6% borderline cases. FNAC demonstrated 93.9% sensitivity, 100% specificity, 100% positive predictive value (PPV), 93.1% negative predictive value (NPV), and 96.7% accuracy. The frozen-section analysis exhibited 96.9% sensitivity, 100% specificity, 100% PPV, 96.4% NPV, and 98.3% accuracy.

Conclusion: Intraoperative frozen-section analysis displays superior diagnostic utility compared to preoperative FNAC. However, histopathology remains the definitive gold standard. Integrating all three diagnostic modalities is crucial for precise diagnosis and effective patient management.

## Introduction

Breast disorders and the prevalence of breast lumps are concerning issues for females, with a notable portion being benign, yet a significant percentage turning out to be malignant lesions. Distinguishing between benign and malignant lumps based solely on clinical evaluation is often challenging, prompting the necessity for a conclusive diagnostic approach to ensure optimal treatment outcomes [1].

Fine-needle aspiration cytology (FNAC) constitutes a fundamental element of the "triple approach" to breast examination, which integrates clinical assessment, radiological imaging, and FNAC. FNAC involves microscopic examination of cellular material collected through a small-gauge needle and a sealed syringe [2]. Its value lies in simplicity, reliability, minimal invasiveness, cost-effectiveness, and freedom from major complications without requiring a cryostat [3]. However, FNAC has limitations, occasionally failing to provide a clear diagnosis due to inherent constraints in cytological examinations or insufficient material [2].

By contrast, the frozen-section method for intraoperative pathological diagnosis has a century-long history. Despite advances in cytological techniques and increasing pre-surgery core needle biopsies, frozen sections remain crucial diagnostic tools in specific settings. Their primary role is swift intra- or perioperative diagnosis for effective patient management. In addition, they confirm carcinoma diagnosis when FNAC reports are inconclusive and assess resected margins in carcinoma cases [4]. However, freezing artifacts may compromise tissue specimen evaluation. Hence, histopathological examination remains a dependable resource for surgeons and oncologists, guiding contemporary therapeutic decisions [1].

This study is undertaken to assess the utility of both FNAC and frozen-section procedures in a series of patients presenting with breast lumps. This study aims to analyze the morphological characteristics of benign and malignant breast lesions using FNAC, frozen sections, and histopathology and evaluate the sensitivity, specificity, and predictive values of FNAC and frozen sections against histopathology as the reference standard. By doing so, we wish to identify a diagnostic modality that can assist healthcare professionals in both diagnosing conditions and determining treatment outcomes tailored to the specific study population, and we seek to contribute valuable insights into the diagnostic efficacy of these methods in the context of breast lesions, ultimately improving patient care and optimizing treatment strategies.

## Materials and methods

A descriptive cross-sectional investigation was conducted from November 2017 to August 2019 at KVG Medical College and Hospital, a tertiary care multi-specialty hospital situated in Rural Karnataka, a state in South India, with prior approval from the institutional ethics committee (IEC: 26.10.2017-1). The study focused on female patients seeking consultation at the Pathology Department specifically for breast lesions. All the patients who came to the department during the study period and met the inclusion criteria were part of the study. The final sample size was 60.

The inclusion criteria encompassed female patients diagnosed with breast lumps or lesions through clinical and radiologically by USG and mammography, followed by FNAC and subsequent excision biopsy or mastectomies. The exclusion criteria involved male patients with breast lumps, individuals undergoing chemotherapy, and those without histopathological correlation after FNAC. The FNAC procedure involved obtaining written informed consent, using disposable syringes for aspiration, targeting palpable lesions, collecting cellular material on slides, wet fixation in 95% methanol, and staining with hematoxylin and eosin (H&E) or Leishman stain within an hour.

Sample adequacy was ensured with criteria including at least six clusters of ductal cells, each containing at least 10 cells, and multiple passes from different angles of the lump. Cytology reporting followed the International Academy of Cytology (IAC) guidelines [5]: C1 (insufficient material), C2 (benign), C3 (atypical), C4 (suspicious for malignancy), and C5 (malignant). Frozen sections were performed on freshly received breast lesion specimens. Tissue sections were mounted on metallic holders with optimal cutting temperature compound (OCTC) and rapidly frozen to around -200 to -300°C. The sections were cut with a cryostat, placed on glass slides, and stained with rapid H&E. Frozen-section slides were immediately examined and reported as benign, malignant, or inconclusive, following routine histopathology criteria. Reporting of histopathology adhered to the WHO classification of breast tumors (12th edition) [6]. FNAC and frozen-section diagnoses were correlated with histopathological reports for the final diagnosis.

Statistical analysis involved data collection and entry in Microsoft Excel 2007 (Microsoft Office, USA), with analysis in SPSS version 16 (released 2007, SPSS for Windows, SPSS Inc., Chicago, USA). Data were tabulated as percentages, mean, and standard deviation. Sensitivity, specificity, positive predictive value (PPV), negative predictive value (NPV), and overall accuracy of the FNAC and frozen sections were calculated and compared with histopathology.

## Results

A total of 60 female patients visiting the Pathology Department with breast lesions participated in the study. Their mean (±standard deviation) age was 41.53 (±16.03) years.

Categorization of breast lesions according to the IAC protocol of FNAC

**Figure 1:**
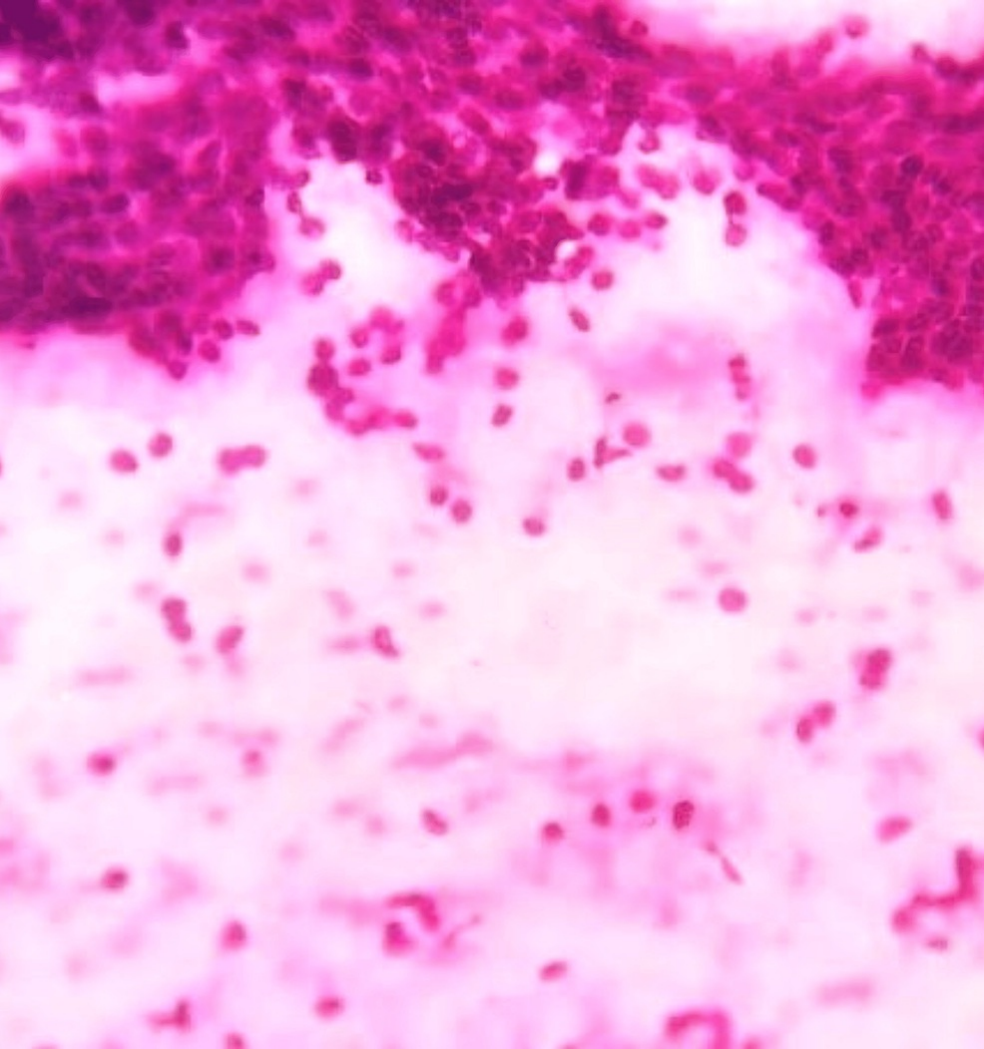
Photomicrograph of fibroadenoma on FNAC showing benign ductal epithelial cells admixed with myoepithelial cells in a background of numerous bare bipolar nuclei (H&E, 10x). FNAC: fine-needle aspiration cytology

**Figure 2:**
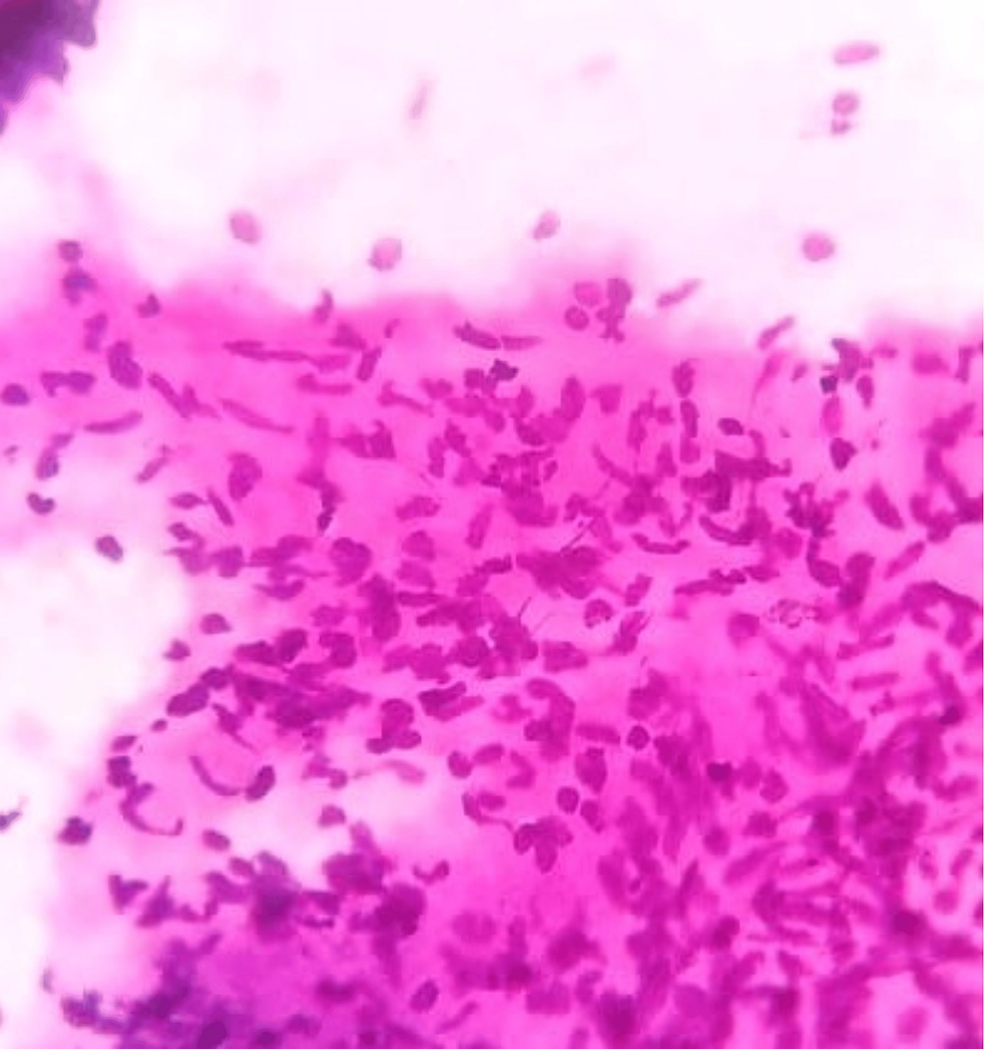
Photomicrograph of Benign phyllodes tumour on FNAC showing cellular dispersed cells with plump and spindle nuclei in a background of fibrous stroma (H&E, 10x) FNAC: fine-needle aspiration cytology

**Figure 3:**
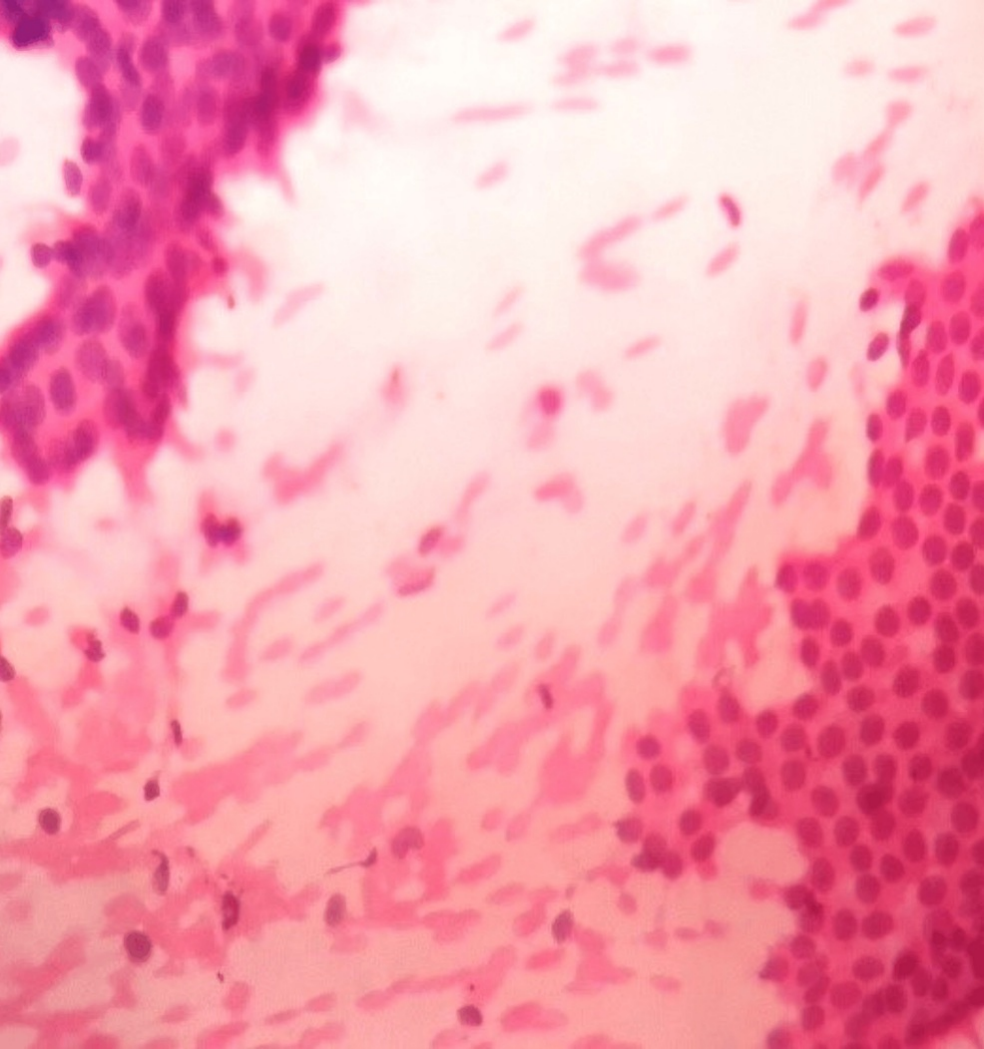
Photomicrograph of fibroadenomatoid hyperplasia of the breast (FHAB)on FNAC showing mild epithelial hyperplasia and clusters of apocrine metaplastic cells (H&E, 10x). FNAC: fine-needle aspiration cytology

**Figure 4:**
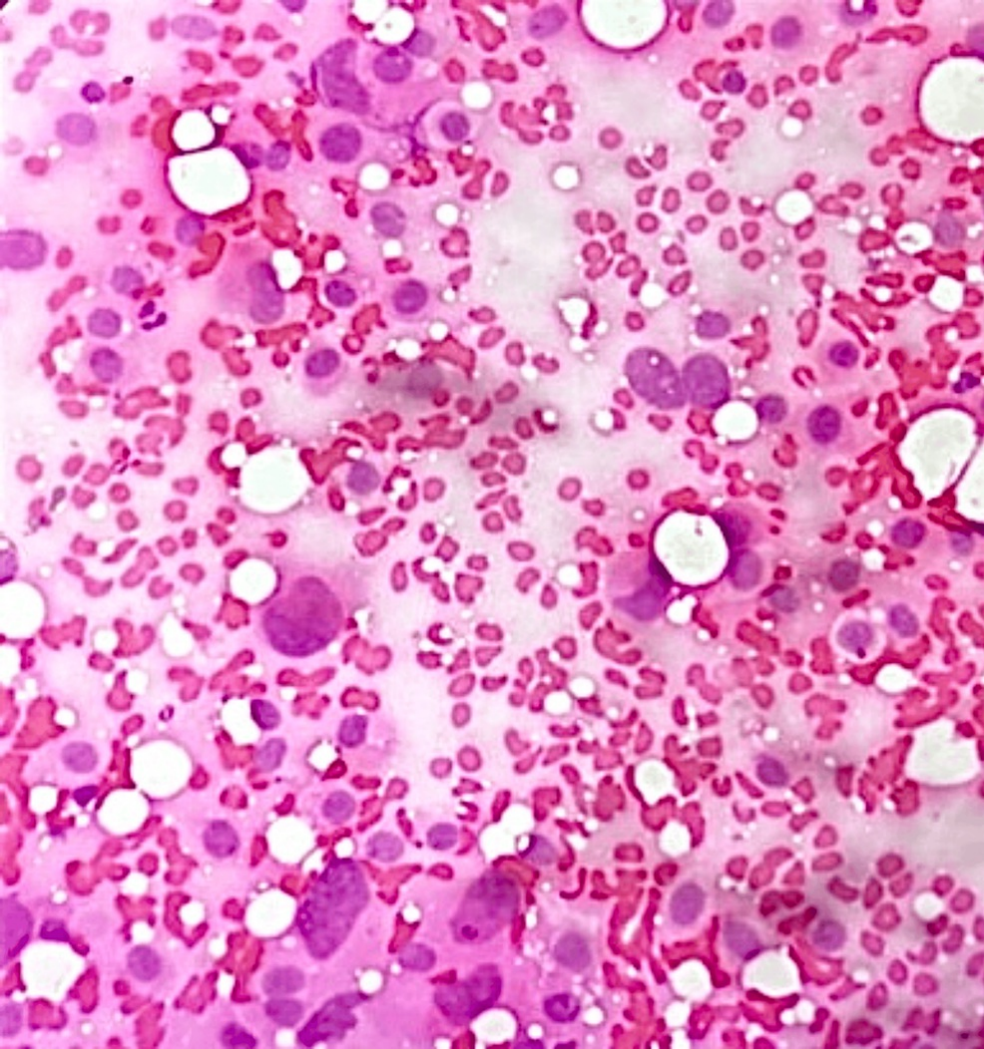
Photomicrograph of borderline phyllodes on FNAC showing moderate cellularity and atypia (H&E, 10x). FNAC: fine-needle aspiration cytology

**Figure 5:**
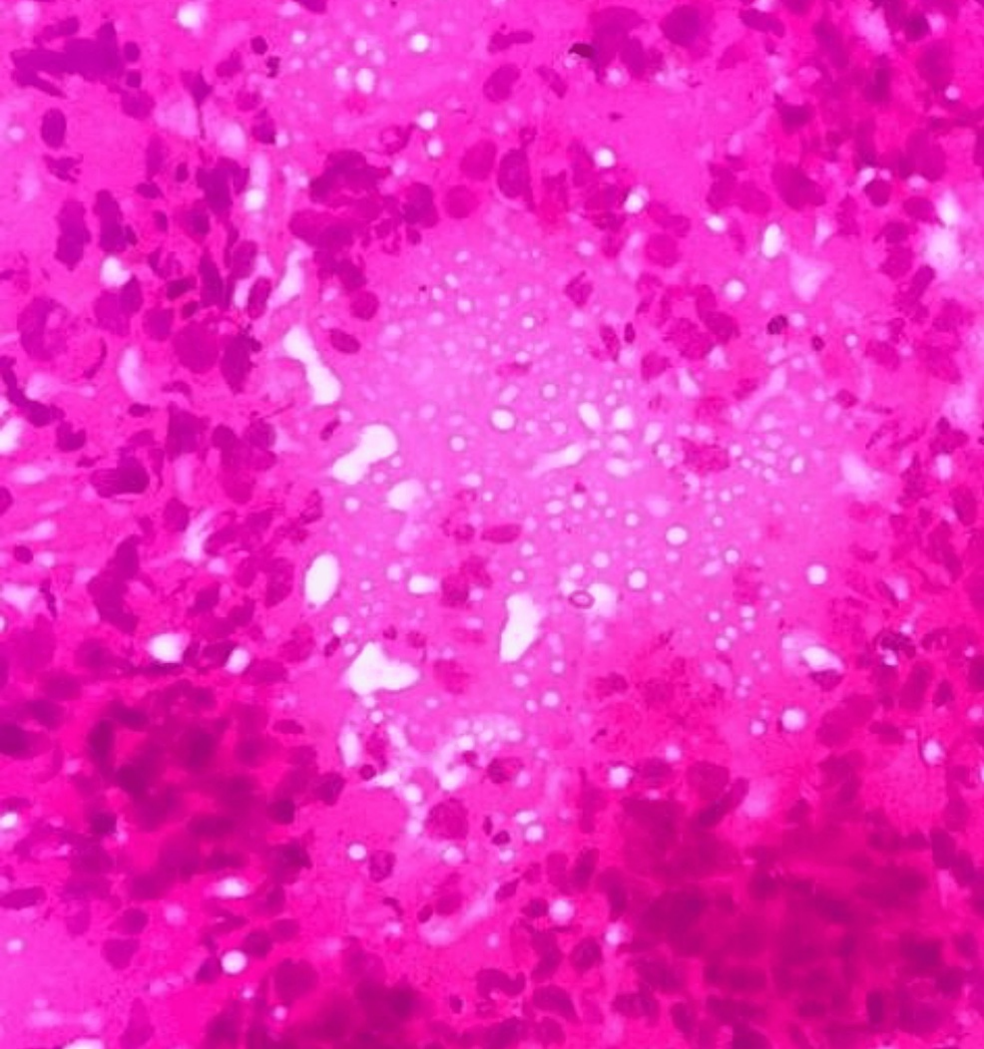
Photomicrograph of malignant phyllodes on FNAC showing cohesive fragments of highly cellular stroma composed of spindle cells with marked nuclear pleomorphism (H&E, 40x). FNAC: fine-needle aspiration cytology

**Figure 6:**
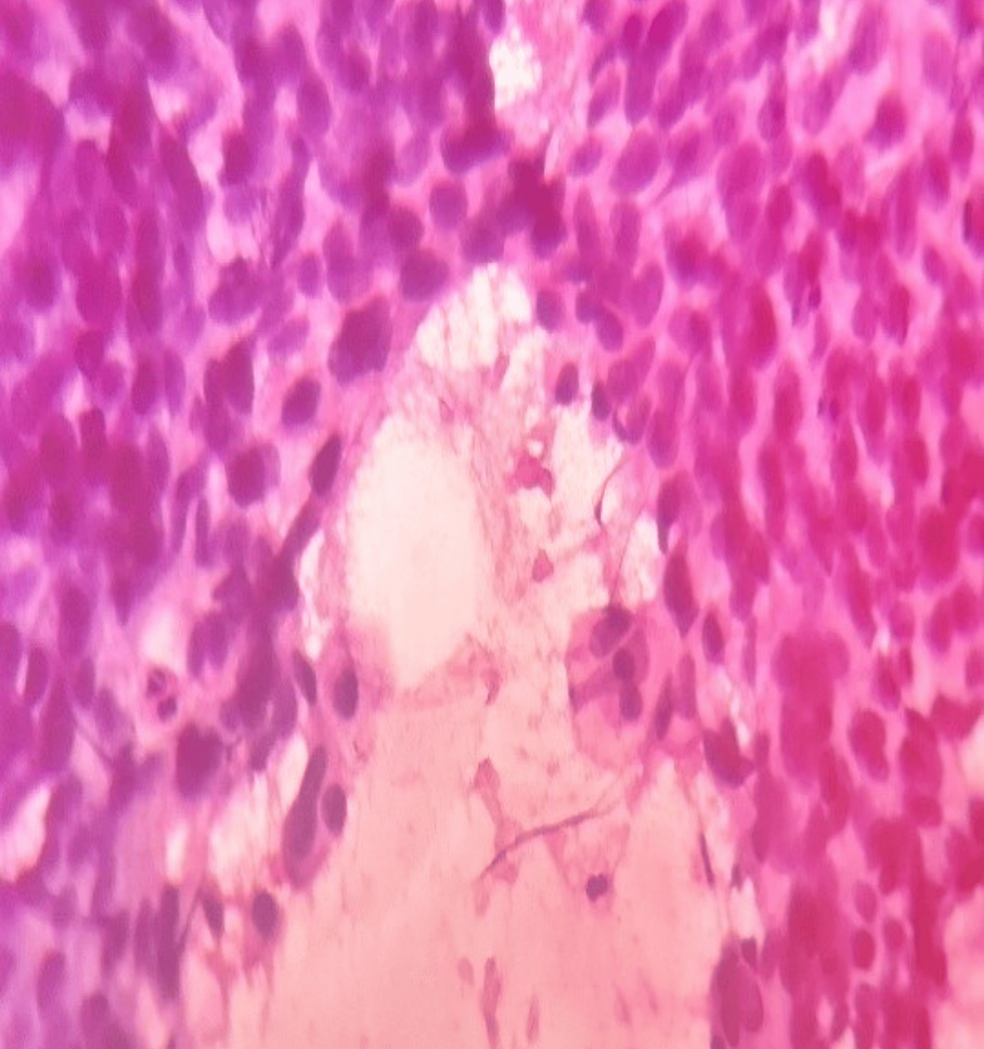
Photomicrograph of FNAC of infiltrating lobular carcinoma showing poorly cohesive clusters, single files of cells having uniformly small nuclei with an irregular membrane (H&E,40x). FNAC: fine-needle aspiration cytology, H&E: hematoxylin and eosin

**Figure 7:**
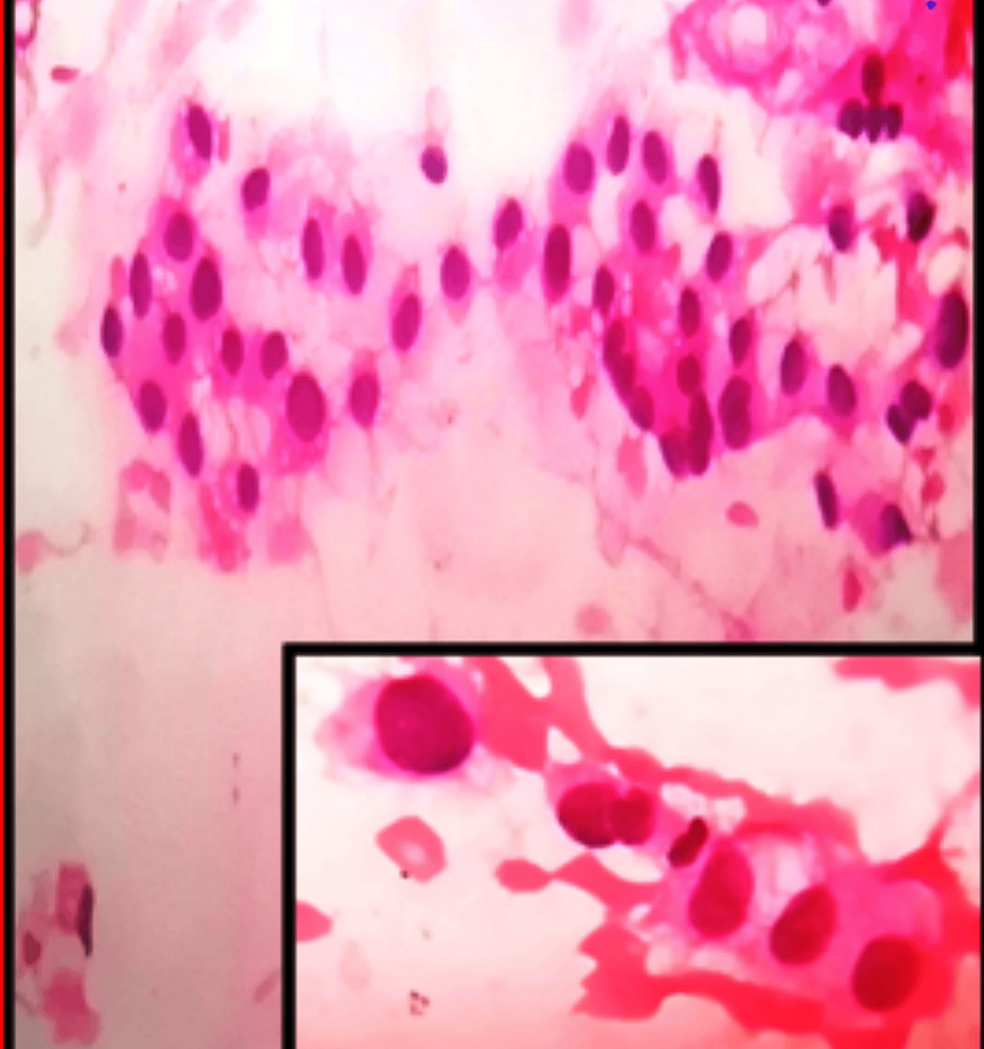
Photomicrograph of FNAC of infiltrating lobular carcinoma showing poorly cohesive clusters, single files of cells having uniformly small nuclei with an irregular membrane (H&E,40x). FNAC: fine-needle aspiration cytology, H&E: hematoxylin and eosin

The FNAC findings were categorized according to the IAC criteria: The C1 category constituted 3.3% of the cases, C2 constituted 41.6%, C3 constituted 3.3%, C4 constituted 8.3%, and C5 constituted 43.3%. Of the total 60 cases, C5 category lesions (26 cases) were the most common, followed by C2 categories (25 cases) when compared to the rest of the categories. Table 1 shows the distribution of breast lesions according to the IAC category on FNAC. Figures 1-7 show the photomicrographs of various breast lesions on FNAC.

**Table 1:** Distribution of breast lesions according to the IAC category on FNAC IAC: International Academy of Cytology, FNAC: fine-needle aspiration cytology

IAC categories	FNAC findings	Number of cases	Percentage
C1 (3.33%)	Inadequate	02	3.33%
C2 (41.6%)	Fibroadenoma	12	20%
	Fibroadenomatoid hyperplasia	07	11.6%
	Benign phyllodes	04	6.6%
	Fibrocystic disease	01	1.6%
	Chronic mastitis	01	1.6%
C3 (3.3%)	Epithelial hyperplasia	02	3.3%
C4 (8.3%)	Suspicious for malignancy	05	8.3%
C5 (43.3%)	Invasive carcinoma breast, non-specific type	12	20%
	Invasive carcinoma, non-specific type with medullary-like features	06	10%
	Medullary carcinoma	05	8.3%
	Malignant phyllodes	01	1.6%
	Papillary carcinoma	01	1.6%
	Invasive lobular carcinoma	01	1.6%
Total		60	100%

Classification of breast lesions based on the frozen section

Following the frozen section, out of a total of 60 cases, 31 cases (51.6%) were diagnosed as malignant lesions and 27 cases (45%) were diagnosed as benign lesions, while two cases (3.3%) were deferred for histopathological diagnosis.

Distribution of Individual Breast Lesions Based on the Frozen Section

**Figure 8:**
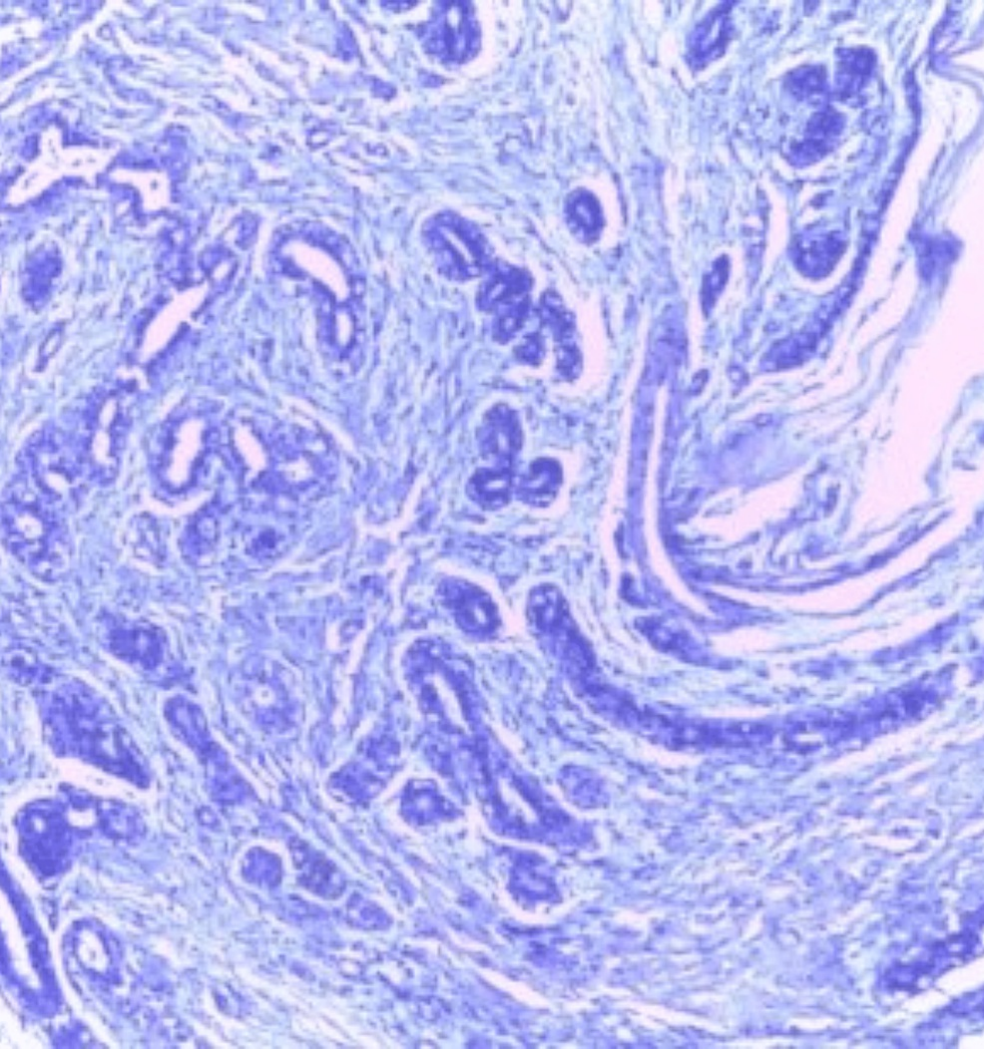
Photomicrograph of fibroadenoma on the frozen section showing intracanalicular and pericanalicular patterns (H&E, 10x)

**Figure 9:**
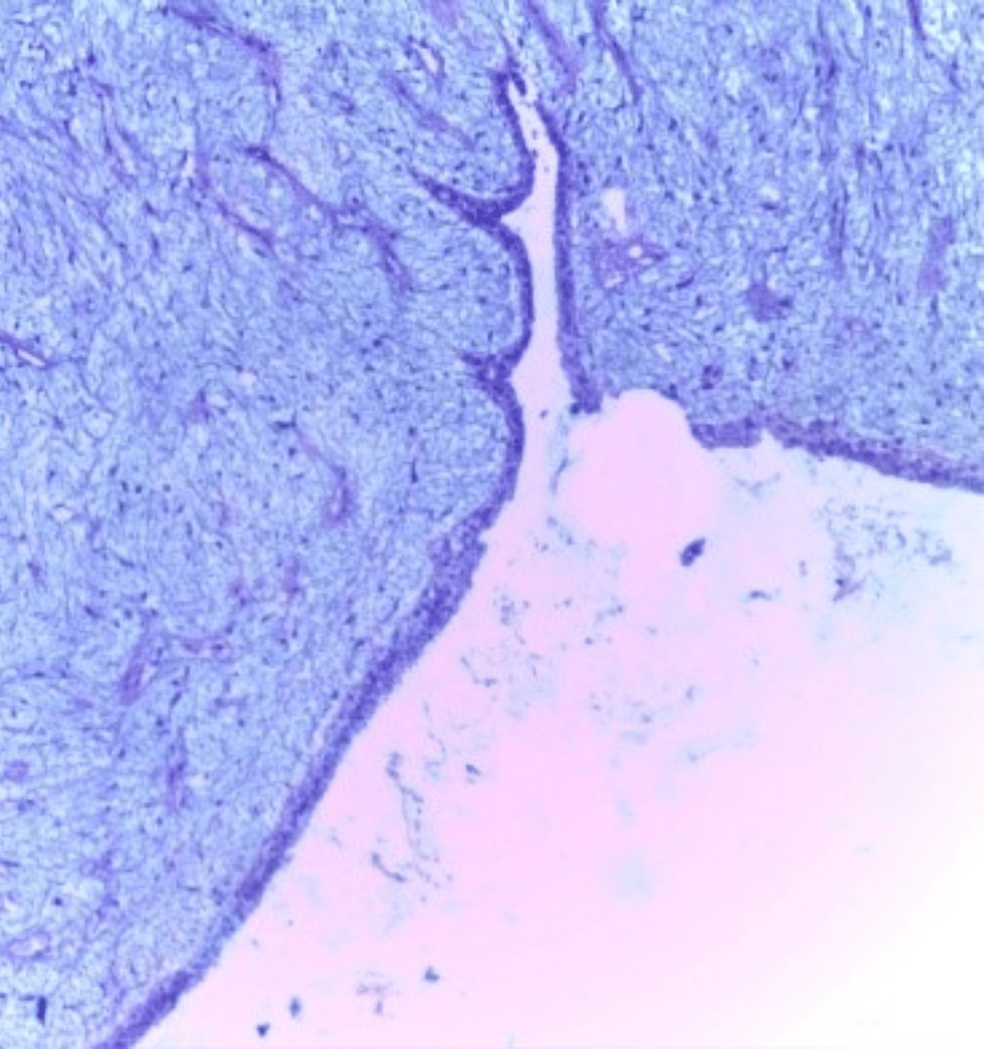
Photomicrograph of a benign phyllodes tumor on the frozen section showing a leaf-like architecture with hypercellular stroma (H&E, 10x).

**Figure 10:**
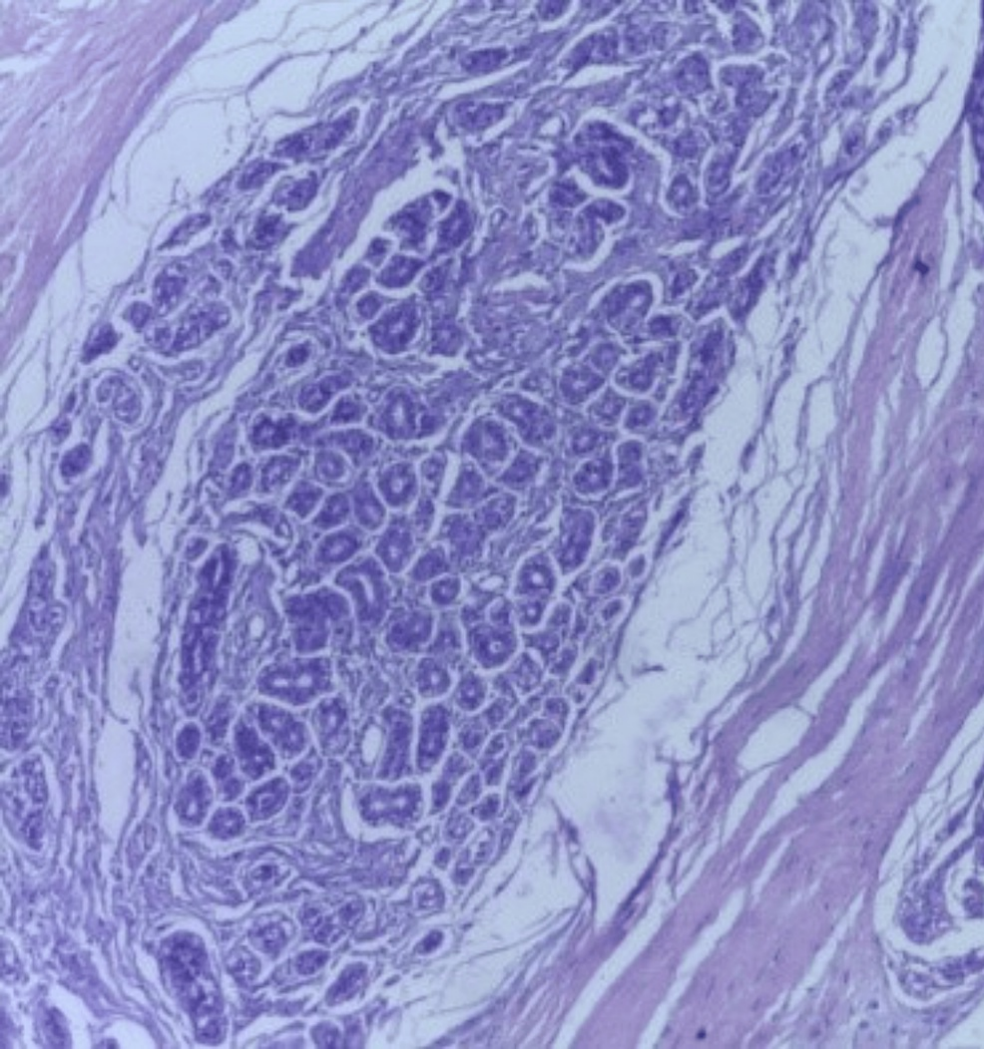
Photomicrograph of fibroadenomatoid hyperplasia of the breast (FHAB) on the frozen section showing proliferation of the intralobular connective tissue (H&E, 10x).

**Figure 11:**
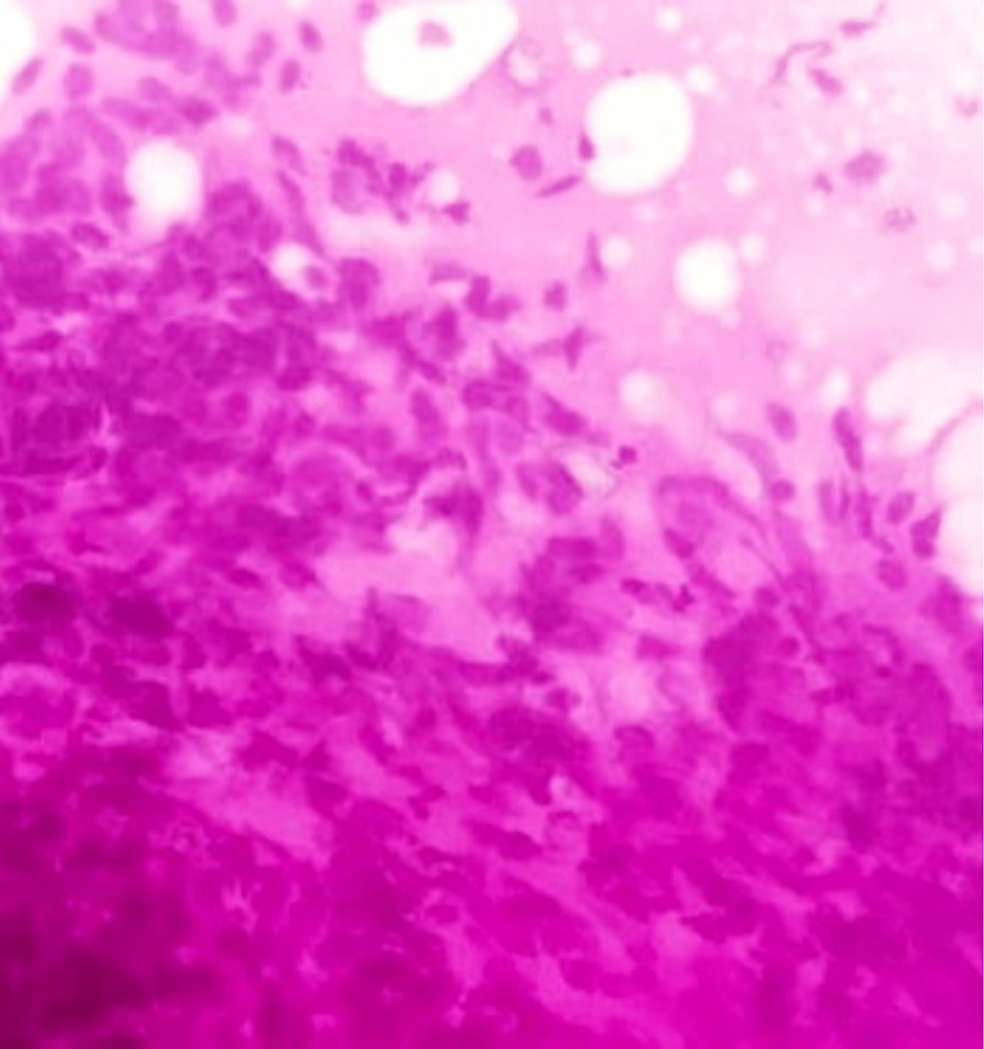
Photomicrograph of borderline phyllodes on the frozen section showing stromal cellularity. The cells have moderate nuclear atypia and frequent mitosis. (H&E, 10x)

**Figure 12:**
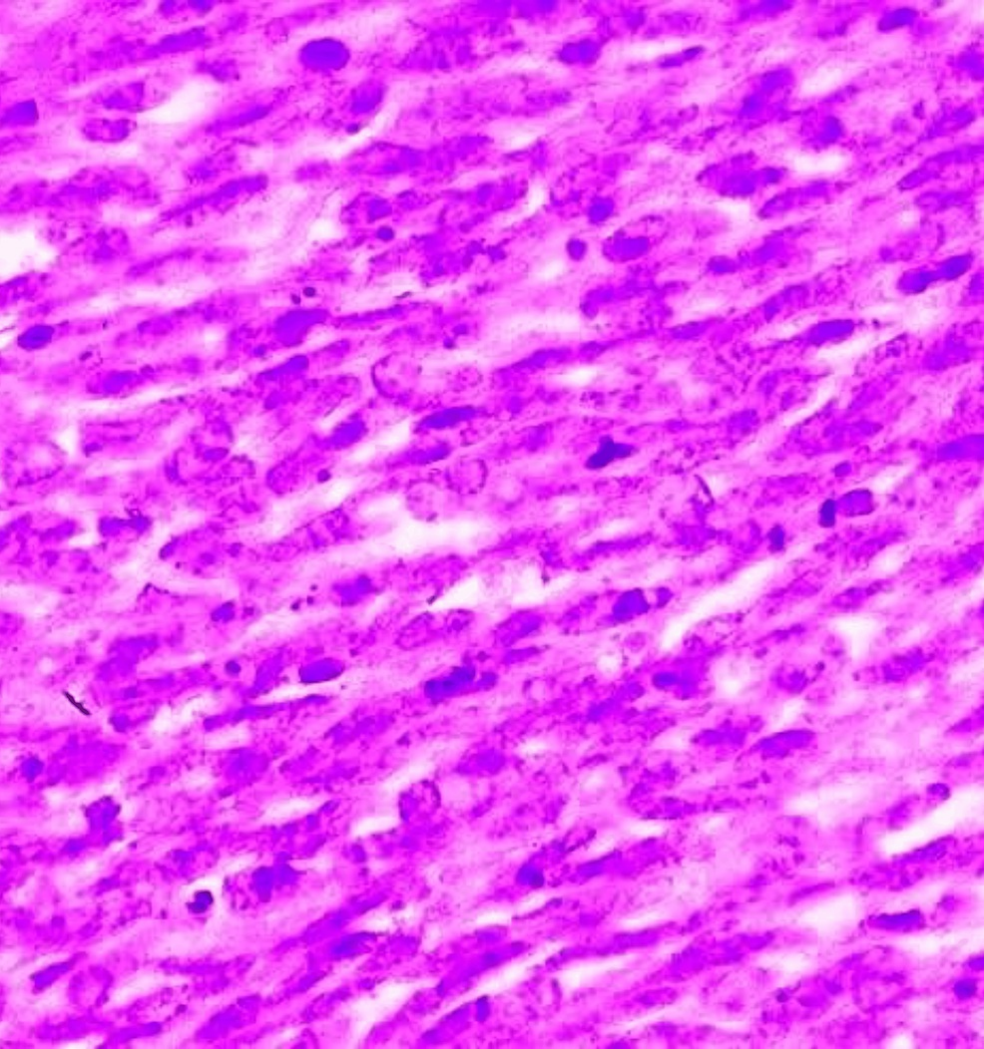
Photomicrograph of malignant phyllodes on the frozen section showing marked stromal cellularity and cellular pleomorphism with numerous mitotic figures. (H&E,40x)

**Figure 13:**
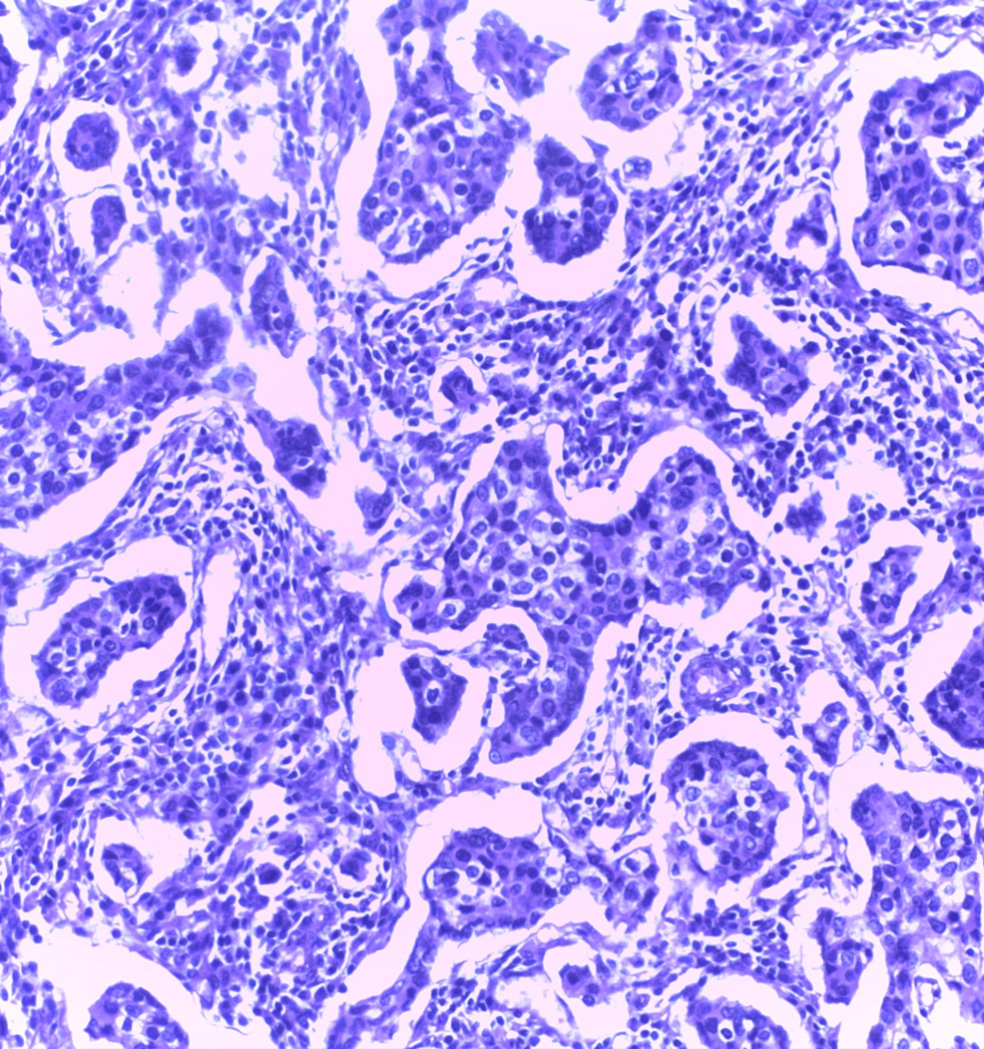
Photomicrograph of invasive carcinoma (no special type) on the frozen section showing tumour cells arranged in cords. The tumor cells have abundant cytoplasm and regular nuclei. (H&E, 40x)

**Figure 14:**
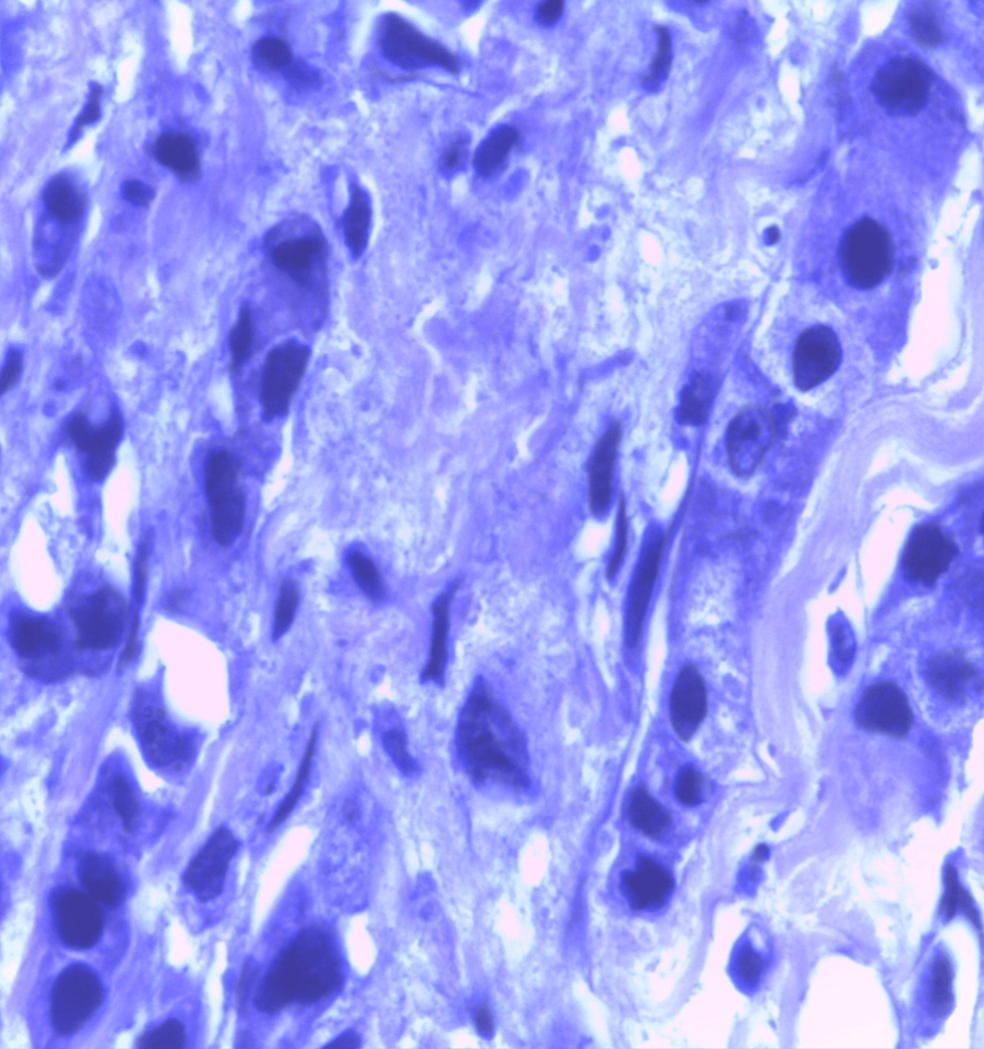
Photomicrograph of the frozen section of invasive lobular carcinoma showing small uniform dispersed cells in single-file linear cords lacking cell-to-cell cohesion. (H&E,40x)

In the present study, out of the 45% of benign lesions, fibroadenoma (20%) was the most common breast lesion, and of the 51.6% of malignant lesions, invasive carcinoma of the breast (non-specific type) was the most common in our study, with 23.3% of cases. A small percentage (3.3%) of the cases were deferred for a paraffin section diagnosis, as shown in Table 2. Figures 8-14 show the photomicrographs of various breast lesions in the frozen section.

**Table 2:** Distribution of individual breast lesions based on the frozen section

Classification based on the frozen section	Frozen findings	Number of cases	Percentage
Benign (45%)	Fibroadenoma	12	20%
	Fibroadenomatoid hyperplasia	09	15%
	Benign phyllodes	04	6.66%
	Epithelial hyperplasia	02	3.33%
Malignant (51.6%)	Invasive carcinoma breast, non-specific type	14	23.33%
	Invasive lobular carcinoma	01	1.66%
	Medullary carcinoma	05	8.33%
	Malignant phyllodes	01	1.6%
	Papillary carcinoma	02	3.3%
	Invasive carcinoma, a non-specific type with medullary-like features	08	13.33%
Deferred (3.3%)	Borderline phyllodes	02	3.3%
Total		60	100%

Classification of breast lesions based on histopathology

**Figure 15:**
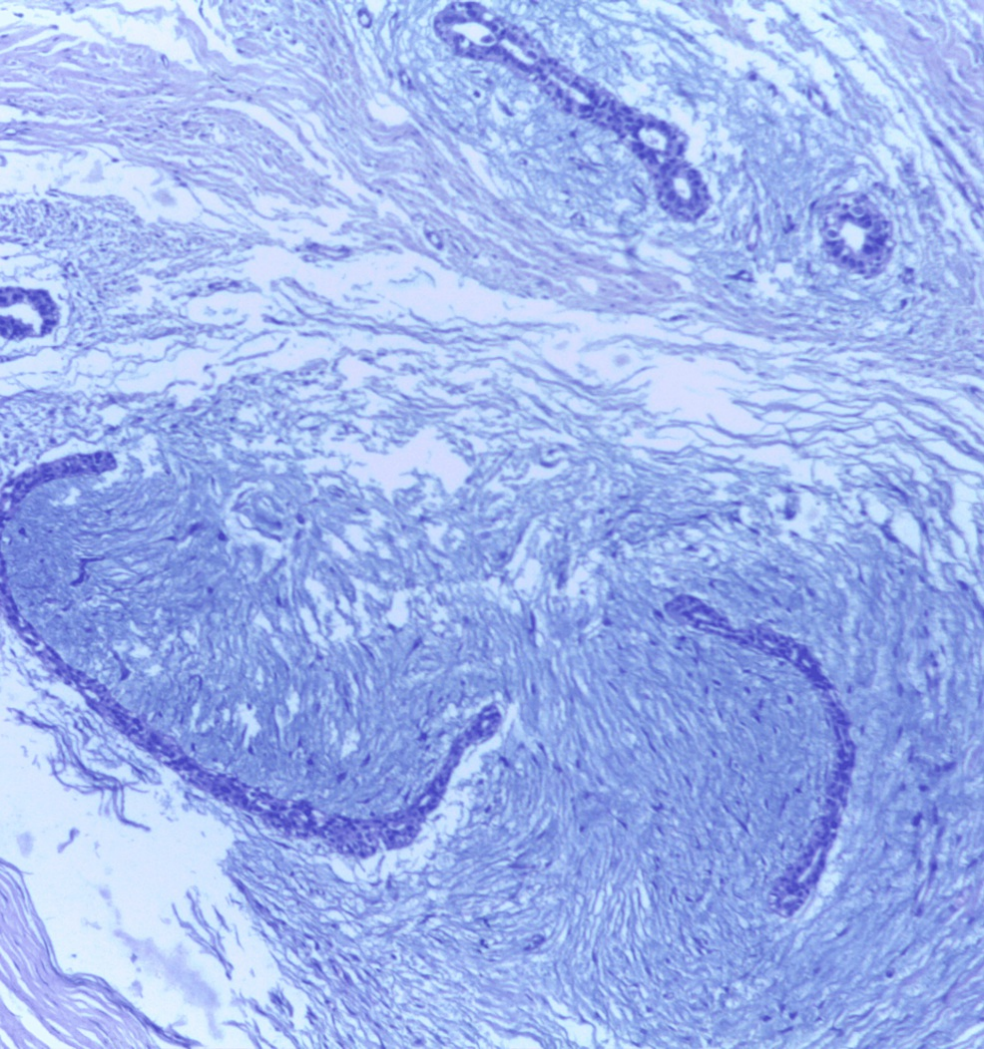
Photomicrograph of fibroadenoma on histopathology showing a pericanalicular pattern in a loose connective tissue stroma (H&E, 10x).

**Figure 16:**
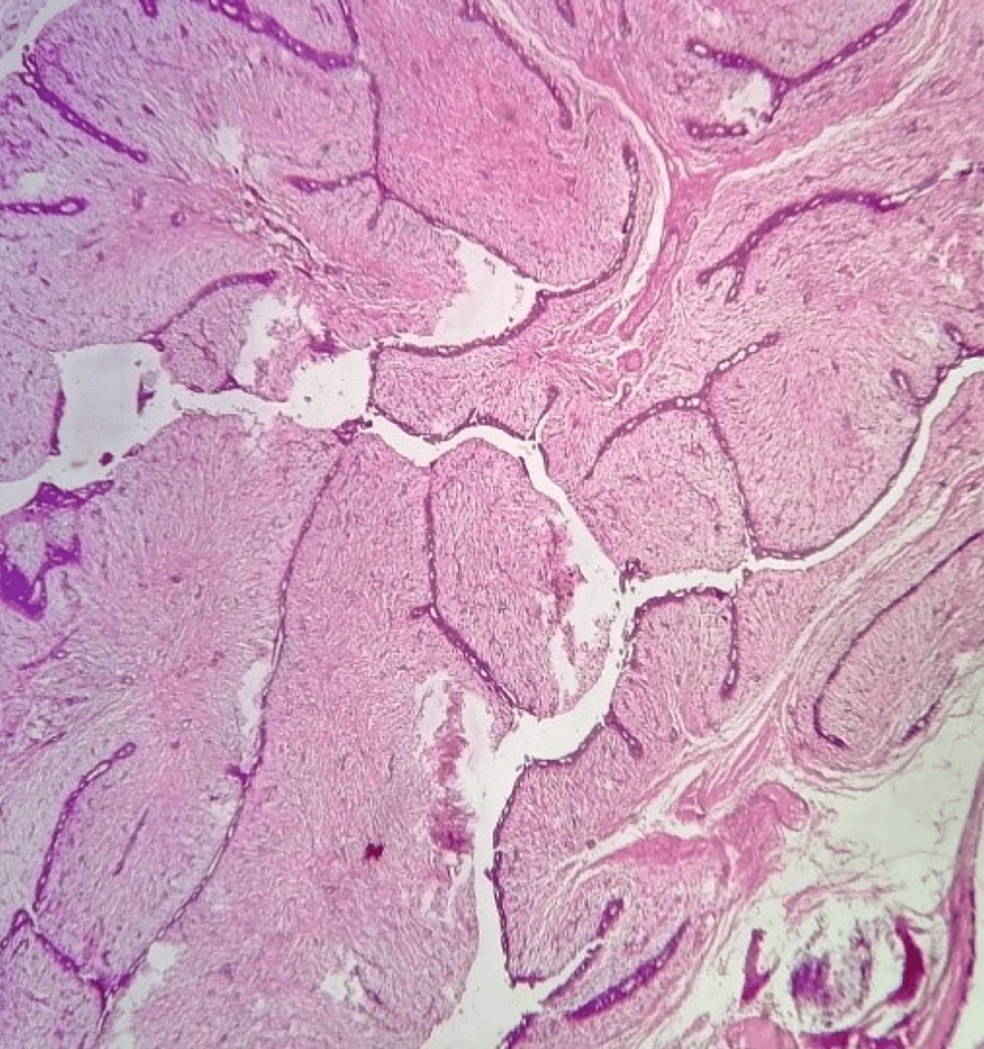
Photomicrograph of benign phyllodes on histopathology showing an enhanced intracanalicular pattern in a cellular stroma (H&E, 10x).

**Figure 17:**
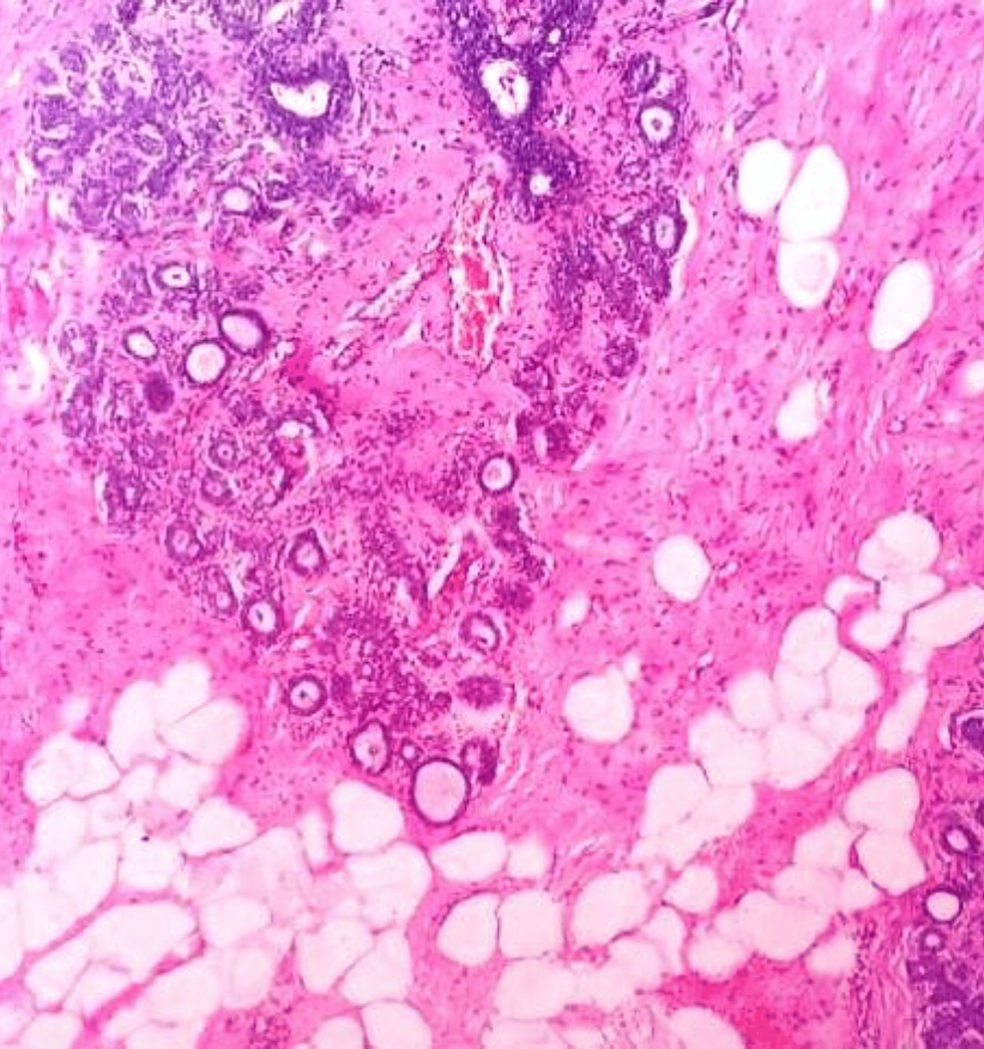
Photomicrograph of fibroadenomatoid hyperplasia of the breast (FHAB) on histopathology showing proliferation of the intralobular connective tissue which resembles fibroadenoma (H&E, 10x).

**Figure 18:**
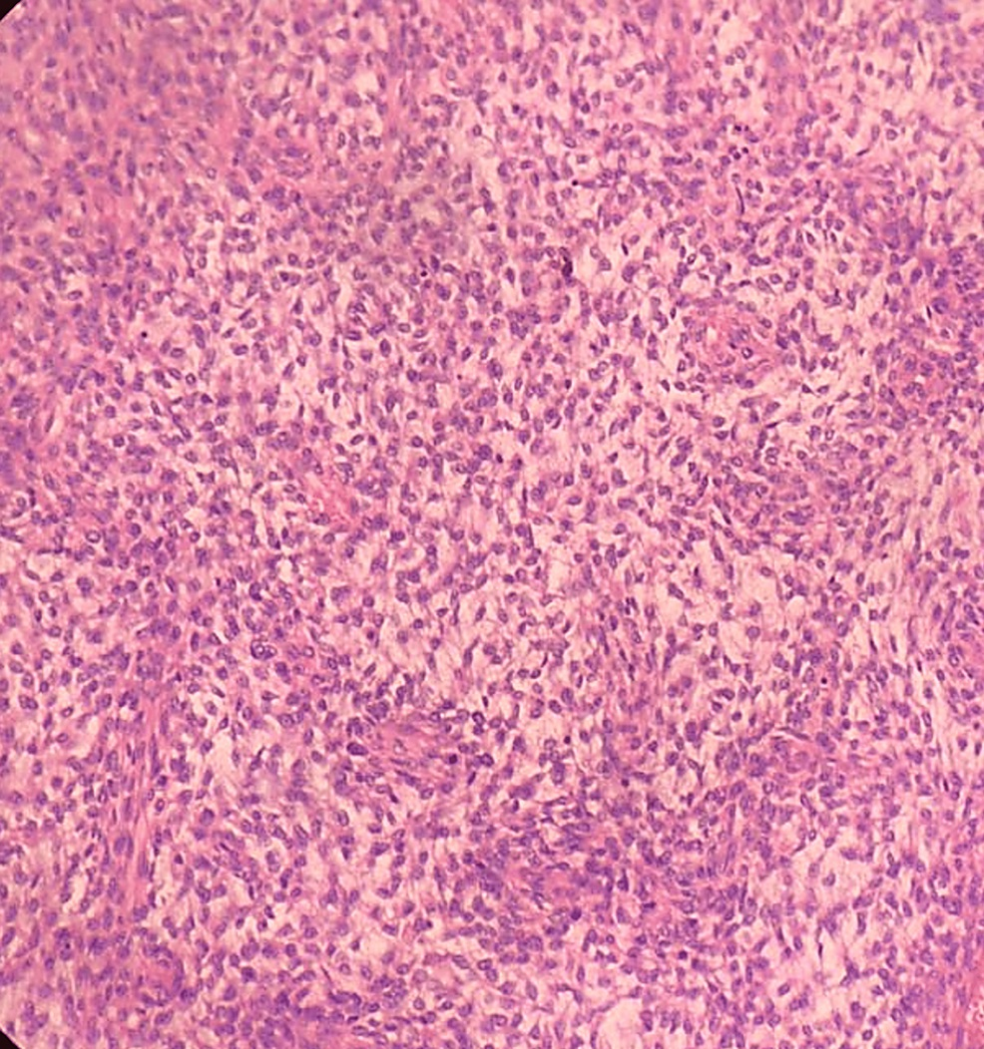
Photomicrograph of borderline phyllodes on histopathology showing stromal cellularity. The cells have moderate nuclear atypia and frequent mitosis. (H&E, 10x)

**Figure 19:**
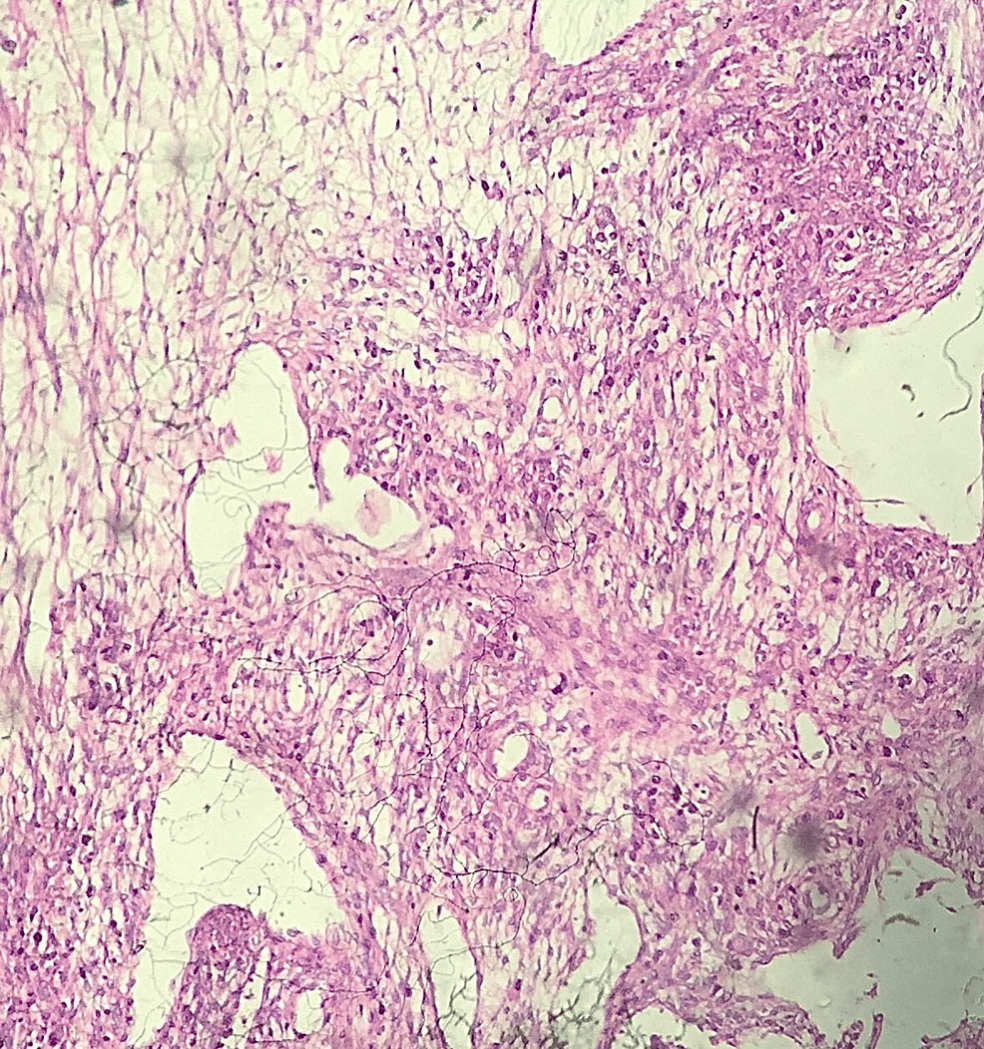
Photomicrograph of malignant phyllodes on histopathology showing marked stromal cellularity, stromal overgrowth, stromal atypia, and abundant mitosis. (H&E, 40x)

**Figure 20:**
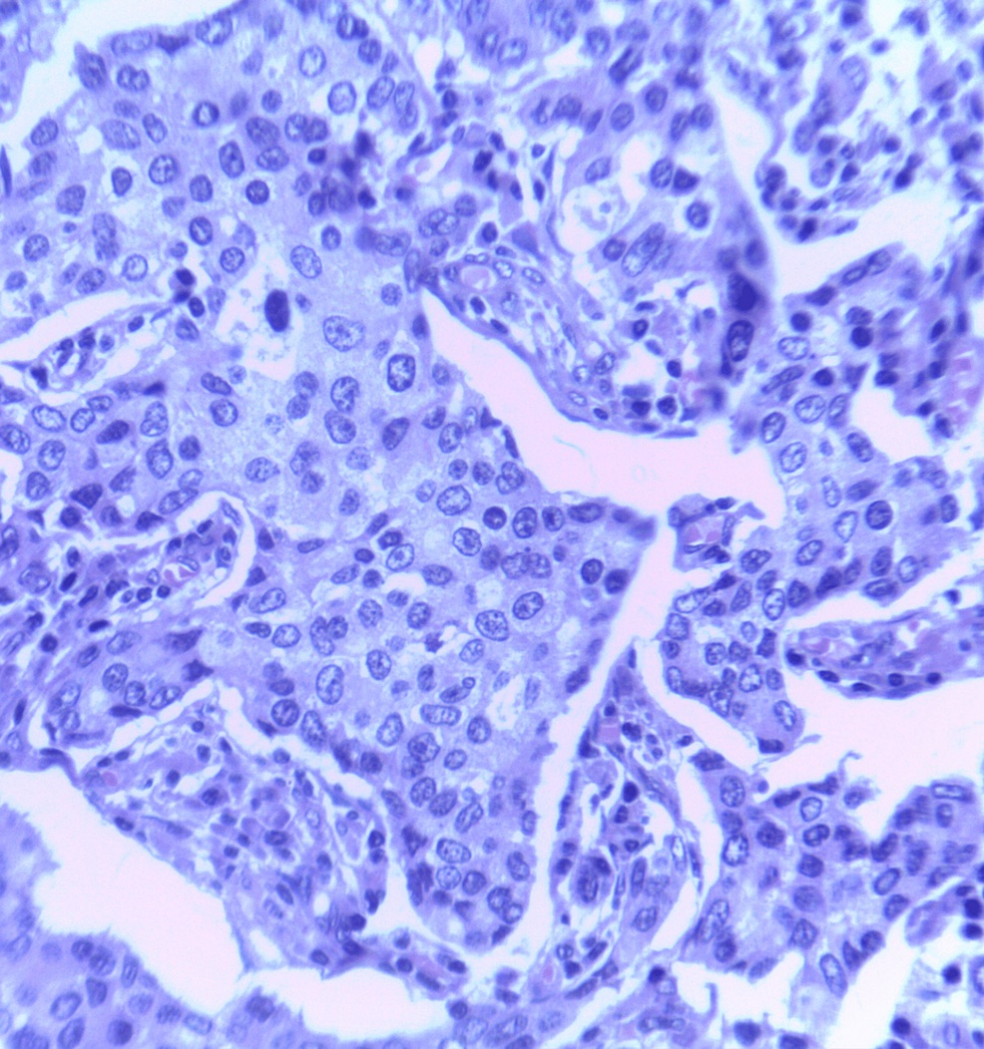
Photomicrograph of invasive carcinoma (no special type) on histopathology showing tumor cells arranged in cords and tubule formation. The cells have abundant cytoplasm with regular nuclei and nucleoli. (H&E,40x)

**Figure 21:**
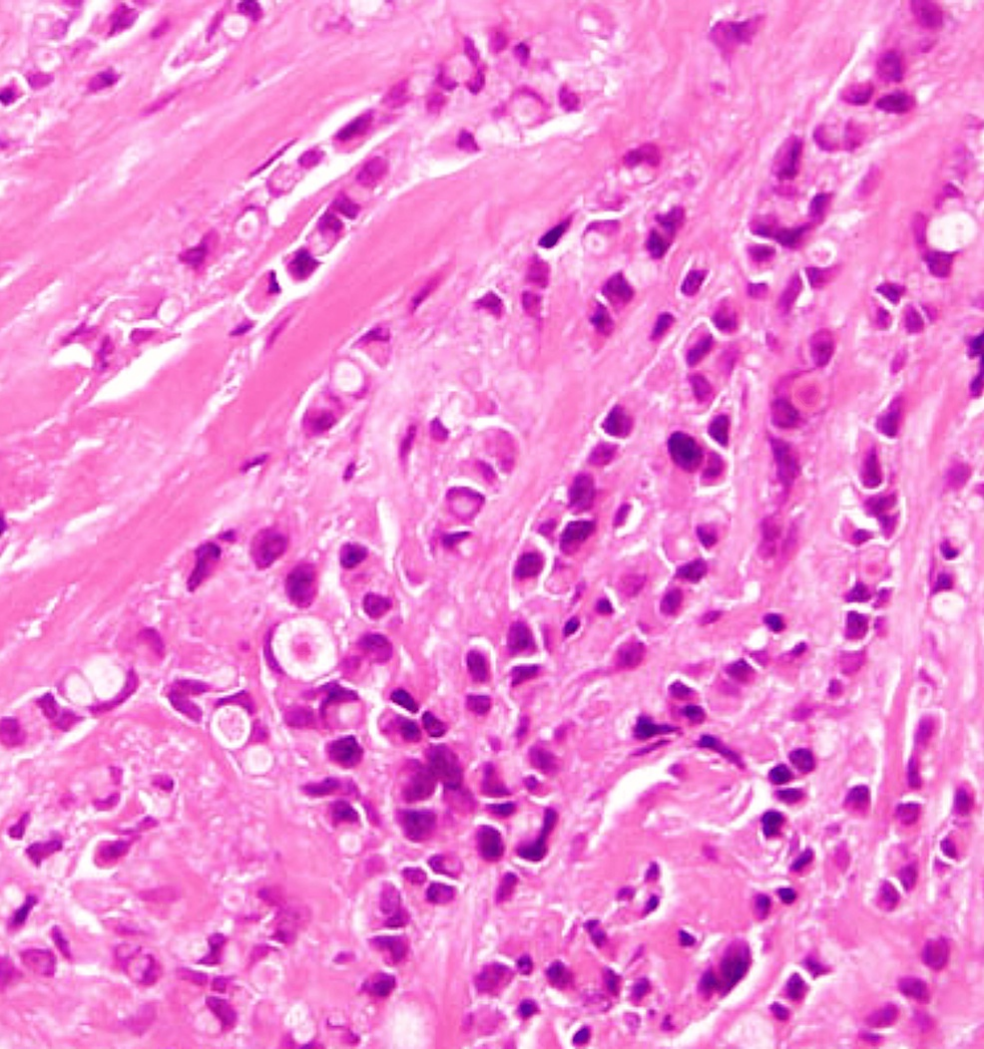
Photomicrograph of the histopathology of invasive lobular carcinoma showing small uniformly dispersed cells in single-file linear cords. The neoplastic cells show an intracytoplasmic lumen. (H&E,40x)

Out of the 60 cases, 32 cases (53.3%) were malignant, 27 cases (45%) were benign, and one case (1.6%) was borderline (borderline phyllodes). All benign cases of the frozen section were diagnosed as benign, and malignant cases of the frozen section were diagnosed as malignant even on histopathology. By contrast, the two deferred cases on the frozen section were diagnosed as borderline phyllodes (one case) and malignant phyllodes (one case) on histopathology. Table 3 shows the distribution of cases based on histopathology. Figures 15-21 show the photomicrographs of various breast lesions on histopathology.

**Table 3:** Distribution of cases based on histopathology

Histopathological findings	Number of cases	Percentage
Benign (27 cases)		
Fibroadenoma	12	20%
Fibroadenomatoid hyperplasia	09	15%
Benign phyllodes	04	6.6%
Epithelial hyperplasia	02	3.3%
Borderline (1 case)		
Borderline phyllodes	01	1.6%
Malignant (32 cases)		
Invasive carcinoma, non-specific type	14	23.3%
Invasive lobular carcinoma	01	1.6%
Invasive carcinoma, non-specific type with medullary-like features	08	13.3%
Medullary carcinoma	05	8.33%
Papillary carcinoma	02	3.33%
Malignant phyllodes	02	3.33%

FNAC diagnosis with histopathological diagnosis

On comparison of FNAC diagnosis with histopathology, among the 60 cases, two cases were found to be inadequate on FNAC, but on histopathology, it was diagnosed as fibroadenoma and fibroadenomatoid hyperplasia of the breast (FAHB), which are benign tumors. A total of 27 cases were diagnosed as benign (C2 and C3) on FNAC, but on histopathology, only 25 cases were found benign, while the remaining two were diagnosed as malignant. Out of the 31 cases categorized as malignancy (C4 and C5) on FNAC, 30 cases were diagnosed as malignant on histopathology. In addition, one case that was initially suspicious for malignancy on FNAC was later categorized as borderline phyllodes upon histopathological examination.

Concordant and discordant cases on FNAC

Out of 60 cases, two FNAC-inadequate cases were labeled benign on histopathology. Among 27 benign FNAC cases, 25 matched histopathology (92.5% concordance), with two discordant cases (chronic mastitis) misdiagnosed as medullary carcinoma and fibrocystic disease misjudged as intracystic papillary carcinoma. All 31 FNAC-malignant cases aligned with histopathology (100% concordance), without any discordant cases. Overall, FNAC exhibited a 96.5% concordance rate and a 3.5% discordance rate, showcasing high consistency between FNAC and histopathology assessments in this study.

Validity of FNAC in diagnosing breast lesions with histopathology as the gold standard

The sensitivity of FNAC in the present study is 93.9%, the specificity is 100%, the PPV is 100%, the NPV is 93.1%, and the accuracy is 96.7%. This confirms that the results of the FNAC findings are significantly matching with the histopathological findings.

Frozen-section diagnosis with histopathology diagnosis

Based on the comparison of the frozen-section diagnosis with histopathology, out of 60 cases, 27 benign cases were benign and 31 cases were malignant both on the frozen section and histopathology. Two cases that were deferred on the frozen section were diagnosed as borderline and malignant lesions on histopathology.

Concordant and discordant cases on the frozen section

Of the 60 cases, 27 cases (45%) diagnosed benign on the frozen section were diagnosed benign even on histopathology, and hence all the benign cases were concordant. There were two deferred cases (3.3%) for histopathological diagnosis on the frozen section because the section revealed worrisome microscopic features having increased cellularity, pleomorphism, and mitosis. On histopathology, among the two deferred cases, one case was diagnosed as borderline phyllodes, while the other case was diagnosed as a malignant phyllodes. Since the suspicion of malignancy was raised on the frozen section, it was considered concordant. Of the 31 malignant cases on the frozen section, all 31 cases were diagnosed as malignant even on histopathology and hence were concordant. There were no discordant cases in the frozen section. The concordance rate of the frozen section for benign and malignant lesions was 100%.

Validity of frozen section in diagnosing breast lesions with histopathology as the gold standard

The sensitivity of the frozen section in the present study is 100% sensitivity, specificity is 100%, the PPV is 100%, the NPV is 100%, and accuracy is 100%. This confirms that the frozen-section findings are significantly matching with histopathological findings. 

## Discussion

Breast cancer is a major concern globally, and while preventing it remains challenging, early detection significantly reduces mortality. FNAC is an established method for accurately characterizing breast lumps, aiding investigation, and informing preoperative decisions. Intraoperative frozen section tests distinguish benign and malignant lesions. The present study evaluates FNAC and frozen section's diagnostic value, correlating with histopathology for breast lesion assessment.

Reporting protocol for FNAC of the breast

In this study, among the five IAC categories [5], C5 had the highest frequency of cases, followed by C2 and then C4. The higher incidence of C5 (malignant) breast lesions might be attributed to increased risk factors among different ethnicities, ages, and patient awareness, as many presented with long-standing breast issues.

Comparative analysis of breast lesions based on IAC categories in various studies

In the present study, insufficient cases were 3.3%, benign cases 41.6%, atypical and probably benign cases 3.3%, suspicious cases 8.3%, and malignant cases 43.3%, whereas Montezuma et al. [7] observed 5.77% insufficient cases, 73.3% benign cases, 13.74% atypical probably benign cases, 1.57% suspicious cases, and 5.54% malignant cases.

Distribution of breast lesions based on the IAC categories

C1 Category

In the present study, 3.3% were inadequate cases. Yalavarthi et al. [8] reported 17.66% (59 cases) inadequacy in aspirations. Bibbo et al. [9] found 13.2% inadequacy in FNAC for nonpalpable masses. In our study, inadequate aspirates might stem from deep tumor localization in the breast and patient reluctance for repeat or guided FNAC.

C2 Category

In the current research, among 25 (41.6%) benign cases, fibroadenoma was predominant with 12 cases (20%), followed by fibroadenomatoid hyperplasia with seven cases (11.6%). Chronic mastitis and fibrocystic disease were seen in one (1.6%) case each. Benign phyllodes appeared in four (6.6%) cases. Ishita et al. [10] found fibrocystic disease as the leading benign lesion, followed by fibroadenoma in their study.

C3 Category

In our research, 3.3% of cases fell into the C3 category, all exhibiting epithelial hyperplasia. To avoid overdiagnosis, we cautiously placed these cases in this category, recommending excision biopsy for further analysis. Literature notes C3 percentages ranging from 3.7% to 5.9% of FNAs, where 16% to 52% of C3 cases turn malignant. Arul et al. [11] observed 28 cases (3.8%) in 728 cases.

C4 Category

The C4 category implies a high likelihood of malignancy but lacks the definitive nature of C5. Our study found 8.3% of cases with suspicious features. The combined equivocal diagnostic categories (C3 and C4) accounted for 11.6%, aligning with reported ranges of 4-17.7% by Nguansangiam et al. [12], suggesting appropriate use in our study.

C5 Category

Within the C5 category's 43.3% malignant cases, the most prevalent was invasive carcinoma of the breast (no special type) (20%), followed by invasive carcinoma of the breast with medullary-like features (10%). Medullary carcinoma of the breast comprised 8.3%, while papillary carcinoma, malignant phyllodes, and infiltrating lobular carcinoma each constituted 1.6%. Patel et al. [13] similarly reported 43.3% of malignant cases in their study, resembling our findings.

Correlation of breast lesions based on FNAC and histopathological diagnosis

In our research, within the C1 category, both cases (3.3%) exhibited benign lesions upon histopathological examination: one fibroadenoma and the other fibroadenomatoid hyperplasia. Within the C2 category, 25 cases (45%) displayed benign lesions, while two cases (3.3%) revealed malignancy in histopathological correlation. Among the C3 cases, two cases (3.3%) unveiled benign lesions on histopathology. Zardawi et al.'s [14] study similarly found benign proliferative lesions and low-grade cancers within the C3 category, mirroring our findings. In the C4 category, five cases (8.3%) indicated malignancy on histopathology. Studies by Deb et al. [15] and Yeoh et al. [16] observed 81-97% malignancy in the C4 category, aligning closely with our study. Overall, in our study, 31 cases (51.7%) with malignancy on FNAC demonstrated corresponding features on histopathology.

Distribution of concordant and discordant cases on FNAC

All FNAC-diagnosed malignancies aligned with histopathology. In our study, the concordance rates were 92.5% for benign lesions and 100% for malignancies. Zajdela et al. [17] achieved 89.2% and 88% cytohistologic concordance for benign and malignant lesions, respectively.

Discordant Case on FNAC

Two cases exhibited discordance: one (1.6%) was initially identified as having fibrocystic disease. The patient, experiencing breast pain and a lump, underwent FNAC due to a Breast Imaging Reporting and Data System (BI-RADS) category III imaging report. FNAC revealed necrosis, acute inflammation, cyst macrophages, and scattered bipolar cells. A frozen section during surgery disclosed a papillary carcinoma. Histopathology confirmed cystic spaces with papillary projections and invasive ductal cells. The overall sensitivity of cytological diagnosis of papillary carcinoma was 42% in a study by Prathiba et al. [18]. Michael et al. [19] reported a pseudopapillary structure in fibroadenoma and invasive ductal carcinoma. The other case diagnosed as chronic mastitis turned out to be medullary carcinoma on histopathological examination. FNAC depicted lymphoplasmacytic infiltrates obscuring scanty ductal cells, misinterpreted as chronic mastitis due to a recent fever history. A lumpectomy was planned with a frozen section confirming a medullary lesion. Histopathology aligned with this diagnosis. Sajjan et al. [20] noted a higher lymphocytic cell-to-tumor cell ratio in medullary carcinoma breast. They highlighted the differential diagnosis of chronic mastitis but emphasized the absence of atypical tumor cells. In our study, potential misinterpretation could have occurred due to a single passage during FNAC.

Validity of FNAC with histopathology

In the present study, FNAC had a sensitivity of 93.9%, specificity of 100%, PPV of 100%, NPV of 93.1%, and accuracy of 96.7%. Similar results were observed in a study by Shah et al. [21], with 92.11% sensitivity, 96.88% specificity, 97.22% PPV, 91.18% NPV, and 94.29% accuracy. Bukhari et al. [22] reported 98% sensitivity, 100% specificity, 97% PPV, 100% NPV, and 98% accuracy. Vala et al. [23] found 83.3% sensitivity, 100% specificity, 100% PPV, 92% NPV, and 94.20% accuracy in their research.

Distribution of benign, malignant, and deferred breast lesions based on the frozen section

In our study, out of 60 cases, 27 (45%) cases were benign while two (3.3%) of the cases were deferred and 31 (51.6%) cases were malignant on the frozen-section analysis. Shah et al. [21] found 47.7% benign and 37.14% malignant cases. A similar finding of 55.1% benign lesions and 94.2% malignant lesions was reported by Chaitra et al. [24]. Kalyani and Sultana [25] found 9.09% benign cases and 89.9% malignant lesions in their study.

Distribution of benign and malignant tumors based on the frozen section

Benign Cases

In our study, among 27 cases with benign lesions (45%), fibroadenoma (20%) was the most frequent, succeeded by fibroadenomatoid hyperplasia (13.33%). Benign phyllodes tumor was present in 6.66% and epithelial hyperplasia in 3.3%. Sheikh et al.'s study [21] on 70 cases noted fibroadenoma (18 cases) as the most common among 33 benign lesions, followed by fibrocystic disease (nine cases), epithelial hyperplasia (four cases), and single cases of benign phyllodes and granulomatous mastitis.

Malignant Cases

In our study, among 31 cases (51.6%) of malignant lesions, the most prevalent was invasive carcinoma of the breast (no special type), accounting for 23.33%, followed by invasive ductal carcinoma with medullary-like features at 13.33%. In addition, we observed 8.5% of medullary carcinoma, 3.3% of papillary carcinoma, and 1.6% each of malignant phyllodes and invasive lobular carcinoma. Sheikh et al. [21] reported 37.14% malignancies out of 70 cases, with infiltrating duct carcinoma as the most common (34 cases), along with rare instances of ductal carcinoma in situ (DCIS), infiltrating lobular carcinoma, and malignant phyllodes (1.43% each). Rosen et al. [26] found ductal carcinoma in 75%, lobular carcinoma in 10%, and medullary carcinoma in 9% out of 857 invasive breast carcinoma cases. Sheikh et al. [21] and Niuyun et al. [27] noted malignant phyllodes in the 40-49 age group, coinciding with our findings of two borderline (3.3%) and one malignant phyllodes (1.6%) in the 45-55 age bracket.

Deferred Cases

In our research, 3.3% of the cases were deferred, with one (1.6%) diagnosed as borderline phyllodes and the other as malignant phyllodes. These cases were deferred due to microscopic features showing increased cellularity and mitosis, prompting a need for paraffin section analysis. The operating surgeon was informed of the potential diagnoses. Vaanika et al. [28] reported a 3.5% deferral rate, while Kalyani and Sultana et al. [25] noted 2.19%. Rosen et al. [26] observed a 5.4% deferral rate among 556 consecutive breast biopsies in their study.

Distribution of concordant and discordant cases on frozen sections

In our current research, we observed complete agreement (100% concordance) without any disagreement in the frozen section analysis, mirroring Santana et al.'s study [29] where they reported a 99% concordance rate and only 1% disagreement among 101 breast specimens.

Validity of the frozen section with histopathology

In the present study, sensitivity was 94.1%, specificity was 100%, PPV was 100%, NPV was 92.7%, and accuracy was 96.7%, which are similar to those in the study done by Haeri et al. [3], Kayani and Sultana [25], and Shah et al. [21].

## Conclusions

The present study highlights FNAC's pivotal role in diagnosing breast lumps early, boasting impressive metrics: 93.9% sensitivity, 100% specificity, 100% PPV, 96.7% accuracy, and 93.1% NPV. Widely adopting FNAC in clinical practice alongside histological diagnosis can expedite patient treatment. In addition, the frozen-section analysis significantly reduces the need for further surgeries, showing potentially better metrics than FNAC. However, histopathological diagnosis remains the gold standard. We recommend using FNAC, frozen sections, and histopathology together, alongside clinical and radiological assessment, for a comprehensive diagnosis of suspicious breast lumps, considering the unique strengths and limitations of each diagnostic method.
